# Investigating associations between biting time in the malaria vector *Anopheles arabiensis* Patton and single nucleotide polymorphisms in circadian clock genes: support for sub-structure among *An. arabiensis* in the Kilombero valley of Tanzania

**DOI:** 10.1186/s13071-016-1394-8

**Published:** 2016-02-27

**Authors:** Deodatus Vincent Maliti, C. D. Marsden, B. J. Main, N. J. Govella, Y. Yamasaki, T. C. Collier, K. Kreppel, J. C. Chiu, G. C. Lanzaro, H. M. Ferguson, Y. Lee

**Affiliations:** Environmental Health and Ecological Sciences Thematic Group, Ifakara Health Institute, Ifakara, Morogoro Tanzania; Nelson Mandela African Institute of Science and Technology Tanzania, School of Life Sciences, Arusha, Tanzania; Institute of Biodiversity Animal Health and Comparative Medicine, University of Glasgow, Glasgow, Lancashire UK; School of Veterinary Medicine, University of California Davis, Davis, CA USA; Department of Entomology and Nematology, University of California Davis, Davis, CA USA

## Abstract

**Background:**

There is growing evidence that the widespread use of Long-Lasting Insecticidal Nets (LLINs) is prompting malaria vectors to shift their biting towards times and places where people are not protected, such as earlier in the evening and/or outdoors. It is uncertain whether these behavioural shifts are due to phenotypic plasticity and/or ecological changes within vector communities that favour more exophilic species, or involve genetic factors within vector species to limit their contact with LLINs. Possibly variation in the time and location of mosquito biting has a genetic basis, but as yet this phenomenon has received little investigation. Here we used a candidate gene approach to investigate whether polymorphisms in selected circadian clock genes could explain variation in the time and location of feeding (indoors versus outside) within a natural population of the major African malaria vector *Anopheles arabiensis*.

**Methods:**

Host-seeking *An. arabiensis* were collected from two villages (Lupiro and Sagamaganga) in Tanzania by Human Landing Catch (HLC) technique. Mosquitoes were classified into phenotypes of “early” (7 pm–10 pm) or “late” biting (4 am –7 am), and host-seeking indoors or outdoors. In these samples we genotyped 34 coding SNPs in 8 clock genes (*PER, TIM, CLK, CYC, PDP1, VRI, CRY1, and CRY2*), and tested for associations between these SNPs and biting phenotypes. SNPs in 8 mitochondrial genes (*ATP6*, *ATP8*, *COX1*, *COX2*, *COX3*, *ND3*, *ND5* and *CYTB*) were also genotyped to test population subdivision within *An. arabiensis*.

**Results:**

The candidate clock genes exhibited polymorphism within *An. arabiensis*, but it was unrelated to variation in the timing and location of their biting activity. However, there was evidence of strong genetic structure within *An. arabiensis* populations in association with the *TIM*, which was unrelated to geographic distance. Substructure within *An. arabiensis* was also detected using mitochondrial markers.

**Conclusions:**

The variable timing and location of biting in *An. arabiensis* could not be linked to candidate clock genes that are known to influence behaviour in other Diptera. This finding does not rule out the possibility of a genetic basis to biting behaviour in this malaria vector, but suggests these are complex phenotypes that require more intensive ecological, neuronal and genomic analyses to understand.

**Electronic supplementary material:**

The online version of this article (doi:10.1186/s13071-016-1394-8) contains supplementary material, which is available to authorized users.

## Background

In Africa, the prominent malaria vector species include *Anopheles gambiae* Giles *sensu stricto* (*s.s*.); *Anopheles arabiensis* Patton and *An. coluzzii* Coetzee & Wilkerson are members of the *An. gambiaesensu lato* (*s.l*.) species complex. Females of these species require vertebrate blood to develop their eggs, and regularly feed upon humans in the wild [1]. The stereotypical pattern of host-seeking in these vector species was described in early work by Gillies [2], and is characterised by the onset of a daily period of host-seeking which begins after dusk and increases to a peak around midnight, with 60–80 % of bites estimated to occur between 9 pm and 3 am [3]. This host-seeking activity coincides with the period when most people are indoors and asleep [1, 4]. This pattern of behaviour underlies the success of control measures like Long-Lasting Insecticidal Nets (LLINs) by selectively protecting people when they are asleep at night indoors [5].

However, host-seeking behaviour varies between species within the *An. gambiae* complex. For example, *An. gambiae* (*s.s*.) is highly endophagic (preference to feed indoors) and anthropophagic (preference for biting humans) [6] and feeds predominantly between 9 pm–3 am [7]. Its sibling species *An. arabiensis* can be relatively exophagic and zoophagic (feeding on cattle as well as humans) [6, 8–10], and can spread its biting over a wider period of the night with less distinct peaks in activity [11]. This provides opportunities for vectors like *An. arabiensis* to evade control using LLINs [12].

Within the *An. gambiae* (*s.l*.) species complex, there have been reports of shifts in their behaviours such as increased tendency to feed outdoors [13, 14], to bite earlier or later in the night [15], and reduced anthropophagy [16] in the presence of vector control measures [17–22]. These behavioural shifts have been associated with changes in the species composition towards vector species with more exophilic behaviour in East Africa. Whilst *An. gambiae* s.s. was the most abundant member of the *An. gambiae* (*s.l*.) species complex [23], in some places in Africa this species has significantly declined in association with bed-net use and now *An. arabiensis* constitutes >95 % of the complex [24, 25]. Whilst environmental contributions to malaria vector feeding behaviour have been documented (e.g. increased zoophily and outdoor resting in the presence of livestock [26]), the influence of mosquito genetic factors on their host-seeking activity is poorly understood. Given that the capacity for mosquitoes to rapidly adapt their feeding activity to avoid contact with LLINs [27], there is a need to investigate if this phenomenon has genetic basis and therefore if it can be subjected to selection imposed by mosquito control measures.

The location in which malaria vectors prefer to bite (e.g. indoors or outdoors) has some genetic basis [28]. For example, a chromosome inversion *2Rbc* is associated with outdoor biting and resting behaviour in *An. arabiensis* [29]. Furthermore, the *2Ra* and *3Ra* inversions are associated with endophagy in *An. funestus* [30]. Whilst these studies support the hypothesis that variation in mosquito feeding behaviour is influenced by genetic factors, their use of coarse genetic units such as chromosomal inversions makes it difficult to establish a causal relationship with specific functional genes. The use of more modern, fine-scale genetic analysis approaches based on single nucleotide polymorphisms will enable a much more powerful investigation of phenotype-genotype associations with greater likelihood of identifying the specific genes that influence behaviour [31, 32].

Circadian clock genes are obvious targets for investigation of genes responsible for variation in the timing of daily activity patterns, specifically feeding rhythms in mosquitoes. In *Drosophila, Period* (*PER*) and *Timeless* (*TIM*) have been identified as key clock genes that encode critical components of the molecular oscillator that drives circadian rhythms. *PER* and *TIM* proteins are transcriptional repressors that regulate their own expression through negative feedback mechanisms by suppressing the activity of *CLOCK* [*CLK*] and *CYCLE* [*CYC*], two activators of *PER* and *TIM* transcription [33]). *Vrille* (*VRI*) and *par domain protein 1* (*PDP1*) encodes additional transcription factors with opposing function to control the expression level of *CLK* [34, 35]. *Doubletime* (*DBT*) is also an important clock gene in *Drosophila,* where it encodes a clock kinase that influences circadian rhythmicity through phosphorylation of *PER* protein, thereby setting the speed of the clock [33]. Additional clock genes include *Cryptochrome 1* (*CRY1*), which encodes a flavin binding photoreceptor found ubiquitously in mammals, insects and plants [36], and regulate circadian activities by facilitating the degradation of *TIM* protein *via* the proteasomal pathway in the presence of light [37]. On the other hand, *Crytochrome 2* (*CRY2*), which is only present in some insect species and in mammals, has been shown to encode a transcription factor that replaces PER as a repressor of CLK or BMAL1 protein activity [38]. These genes can therefore serve as a useful starting point for investigating the genetic basis of polymorphisms in the feeding behaviour of malaria vectors.

So far, only a few studies have investigated clock genes in *An. gambiae* (*s.l*.) and their potential association with diel activity such as blood feeding. Previous studies applied transcriptomic approaches to study gene expression patterns [39–41], and established a genome-wide profiling of circadian gene expression in *An. gambiae* (*s.s*.) [42]. However, there has been little investigation of the link between mosquito feeding behaviours and single nucleotide polymorphisms (SNPs) in clock genes occurring in natural populations.

Here, we hypothesise that mutations in clock genes may explain variation in the host-seeking times of African malaria vectors. We used SNPs to test for associations between SNPs in exons of 8 clock genes and the times at which *An. arabiensis* were caught host-seeking within a natural population in Tanzania. We tested whether any observed associations were consistent within mosquitoes attempting to feed indoors and outdoors. We acknowledge that time of feeding can be influenced by many environmental factors such as distance from host, wind direction, wind speed and availability of other hosts. While these variables were somewhat mitigated by conducting all collections within two days per village and in multiple villages, we expect we would be able to detect significant associations if genetic contribution toward the time of feeding phenotype is relatively strong (effect size of 0.6 or greater; see Methods section below for power analysis).

## Methods

### Study sites

**Fig. 1:**
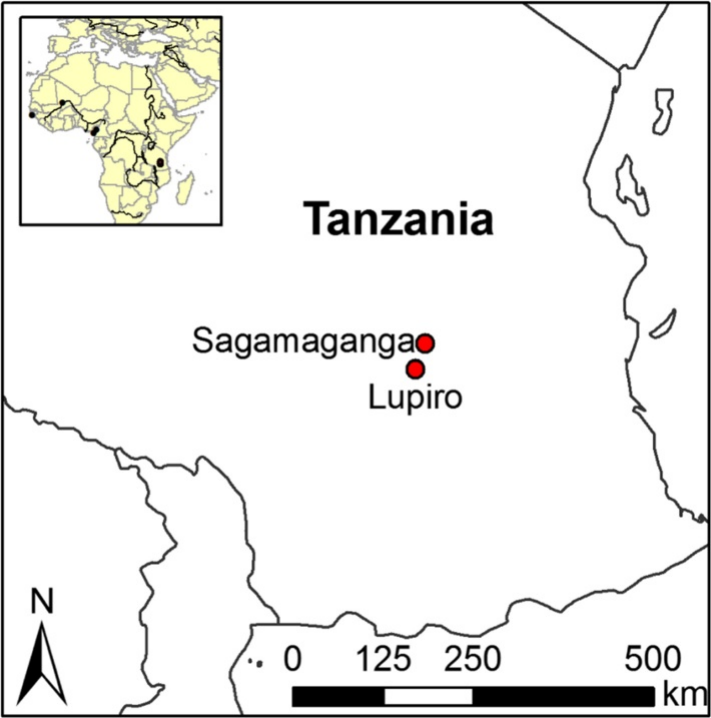
A map of Tanzania showing collection sites. Lupiro and Sagamaganga villages are approximately 40 km away from each other in the highly malaria endemic valley of Kilombero

*Anopheles arabiensis* were collected in the villages of Lupiro (−08.38′S, 36.67′E) and Sagamaganga (−08.07′S; 36.80′E), which are situated about 40 km apart in the Kilombero Valley of Tanzania (Fig. 1). This area has endemic, year-round malaria transmission [43–45] that peaks in the rainy seasons occurring from November to January and from March to May. Residents of Lupiro and Sagamaganga villages are primarily subsistence rice cultivators. Pastoralism is also common in Sagamaganga with many local residents keeping cattle. Both villages have experienced a significant change in the species composition of malaria vectors within recent years as LLINS have reached near 100 % coverage levels.

### Behavioural phenotype selection and mosquito sample collection

Mosquitoes were collected at paired indoor and outdoor trapping stations in three experimental huts per night. Experiments were done between February and March 2013. Mosquitoes were collected by Human Landing Catch (HLC) technique as they attempted to feed on people. These collections were conducted by a volunteer who sat on a chair with his legs exposed from foot to knee. Using a mouth aspirator, the volunteer sucked up mosquitoes as soon as they landed on his exposed legs. Collections were made from 19:00 to 07:00 the next morning, with volunteers actively catching for 45 min in each hour then using the remaining period to rest. Collectors were swapped between different trap types and location of collection to minimise bias that might arise due to differential collectors’ attractiveness to mosquitoes. Captured mosquitoes were placed into holding cups, grouped for each hour of collection. Those morphologically identified as being *An. gambiae* (*s.l*.) were stored in 80 % ethanol to preserve DNA for downstream molecular assays. *Anopheles gambiae* (*s.l*.) specimens were classified into one of four categories based on the time and location they were caught host-seeking: (1) indoor early feeding, (2) indoor late feeding, (3) outdoor early feeding, and (4) outdoor late feeding. Early feeding was defined as mosquitoes collected whilst host-seeking in the early hours of the night (19:00–22:00), while late feeding mosquitoes were those collected whilst host-seeking between 04:00 and 07:00.

### Species identification and SNP discovery analysis

Genomic DNA was extracted from mosquito samples using DNeasy extraction kits (QIAgen, Valencia, CA, USA). First, PCR analysis was performed on specimens to identify their species within the *An. gambiae* (*s.l*.) complex according to Scott’s method [46]. All successfully amplified samples were *An. arabiensis*. This resulted in a final sample size of between 91 and 96 *An. arabiensis* for each phenotype and village combination for genetic analysis. If the phenotype has any genetic basis, we expect to detect association between time of feeding behaviour and genotypes of which effect size is 0.6 or greater with 80 % power given our sample size of N > 90 per group. We used *pwr* library in R software package for power analysis.

Eight clock genes originally identified from the *An. gambiae* (*s.s*.) genome [42] were selected for SNP discovery using conventional Sanger sequencing: *Period* (*PER*)*, Timeless* (*TIM*)*, Clock* (*CLK*)*, Cycle* (*CYC*)*, Pdp1* (*PDP1*)*, Vrille* (*VRI*), *Cryptochrome1* (*CRY1*)*, and Cryptochrome2* (*CRY2*). These genes were selected on the basis of their known association with circadian rhythmic behaviours or function within the molecular oscillator in other insect taxa including *An. gambiae* (*s.s*.) [42, 47, 48]. A series of primers were designed for each gene fragment using Primer3 online tools (http://frodo.wi.mit.edu/primer3/). The identity, specific loci sequenced and primer sequences used for all 8 candidate genes are presented in Table 1.

**Table 1:** Gene and loci identity with the forward and reverse primer sequences

Gene	Gene ID	Loci ID	PCR product size(bp)	Forward primer	Reverse primer
*Clock*	AGAP005711	CLK-E01-267	119	GTAAAATACTCTCCCGGTA	GTAAAATACTCTCCCGGTG
*Clock*	AGAP005711	CLK-E01-192	113	GCTTCGTTCGAGAGAAAGGAA	GCTTCGTTCGAGAGAAAGGAG
*Clock*	AGAP005711	CLK-E01-087	106	CTTGCGCACGGTCGACTTGTCCATC	CTTGCGCACGGTCGACTTGTCCATT
*Clock*	AGAP005711	CLK-E01-240	119	TTCCCGATGATGAACCCGTCC	TTCCCGATGATGAACCCGTCT
*cryptochrome1*	AGAP001958	CRY1-E04-206	120	TCGACGGCGCAGCACGGA	TCGACGGCGCAGCACGGT
*cryptochrome1*	AGAP001958	CRY1-E04-097	99	CGCACGTCCATCGTTC	CGCACGTCCATCGTTT
*cryptochrome1*	AGAP001958	CRY1-E04-240	120	CTACCACCAGCAGCTGTCCA	CTACCACCAGCAGCTGTCCG
*cryptochrome1*	AGAP001958	CRY1-E04-252	102	CGACCTTGACCGACAGTTC	CGACCTTGACCGACAGTTT
*cryptochrome2*	AGAP004261	CRY2-E05-378	113	CCACTGCCATTGCCACCA	CCACTGCCATTGCCACCG
*cryptochrome2*	AGAP004261	CRY2-E05-561	98	GGCGCAGTCGCAGGAAAAC	GGCGCAGTCGCAGGAAAAT
*cryptochrome2*	AGAP004261	CRY2-E05-501	100	TGAGAATGCTGCAGCTGTGAC	TGAGAATGCTGCAGCTGTGAT
*cryptochrome2*	AGAP004261	CRY2-E05-407	113	GCCTTGTTTGGTGTCGTCAGGCA	GCCTTGTTTGGTGTCGTCAGGCG
*cryptochrome2*	AGAP004261	CRY2-E05-045	85	TCCGCTGCCGATGGTC	TCCGCTGCCGATGGTT
*cryptochrome2*	AGAP004261	CRY2-E05-125	82	CCCCAATACCGCACACCGAA	CCCCAATACCGCACACCGAG
*cryptochrome2*	AGAP004261	CRY2-E05-351	102	TATCGTGGGTCCGGGCCGCTA	TATCGTGGGTCCGGGCCGCTG
*cryptochrome2*	AGAP004261	CRY2-E05-051	85	GCGGGAAGCAATCGCA	GCGGGAAGCAATCGCG
*Cycle*	AGAP005655	CYC-E01-034	117	ATTGCTGTTGGAGGGTTTA	ATTGCTGTTGGAGGGTTTG
*Cycle*	AGAP005655	CYC-E01-217	118	CCACTCGTTACACCCTGAGGG	CCACTCGTTACACCCTGAGGT
*Cycle*	AGAP005655	CYC-E01-093	96	GGCAGCGTCCGATTTAAGCCCA	GGCAGCGTCCGATTTAAGCCCG
*Cycle*	AGAP005655	CYC-E01-072	96	GGGTAAAGTGAAGGAGCAACTC	GGGTAAAGTGAAGGAGCAACTG
*Cycle*	AGAP005655	CYC-E01-268	118	ACTTTGCACTTCATCCGA	ACTTTGCACTTCATCCGG
*Cycle*	AGAP005655	CYC-E01-250	118	TGGAAGAAGGAACGGCGC	TGGAAGAAGGAACGGCGA
*Cycle*	AGAP005655	CYC-E01-021	98	TTGATCTTCTTGGGCAGAGC	TTGATCTTCTTGGGCAGAGT
*Pdp1*	AGAP006376	PDP1-E02-110	116	ATCGTCGCGGGACCGCTTC	ATCGTCGCGGGACCGCTTT
*Period*	AGAP001856	PER-PAS-082	101	CGGCTTCCCCAAGGAC	CGGCTTCCCCAAGGAT
*Period*	AGAP001856	PER-PAS-355	100	AGAAGGCGGAGATCATGAGCGGC	AGAAGGCGGAGATCATGAGCGGT
*Period*	AGAP001856	PER-PAS-202	114	GGGGAAAGAGCGGCCAGAAGGAC	GGGGAAAGAGCGGCCAGAAGGAT
*Period*	AGAP001856	PER-PAS-370	100	GGTGGCCGAGATGATC	GGTGGCCGAGATGATG
*Timeless*	AGAP007801	TIM-E05-075	111	GCCCCGTTGACGCTGTCC	GCCCCGTTGACGCTGTCG
*Timeless*	AGAP007801	TIM-E05-087	111	GTATCTGCGTTCCGATGTCG	GTATCTGCGTTCCGATGTCT
*Timeless*	AGAP007801	TIM-E05-189	98	GTCCGCTACGACACAC	GTCCGCTACGACACAT
*Timeless*	AGAP007801	TIM-E05-495	101	CCTACGCTGATTGCCTGGCTA	CCTACGCTGATTGCCTGGCTG
*Vrille*	AGAP007801	VRI-E02-427	118	CCCCGATAAGGATGCGGCCACC	CCCCGATAAGGATGCGGCCACT
*Vrille*	AGAP007801	VRI-E02-355	108	AAGTTGGCGTGCTCGTGA	AAGTTGGCGTGCTCGTGG

PCR products sizes are in base pairs (bp)

Initially, 8 *An. arabiensis* samples from each of the four feeding phenotypes at both sites were sequenced for SNP discovery (*n* = 64). From the 8 genes selected, a total of 34 loci were picked for sequencing. These loci were selected from conservative regions of DNA and included synonymous and non-synonymous mutations that have a codon frequency change of 2 or greater, as is the standard approach to identify mutations that are most likely to influence protein function [49]. The identity of these genes as obtained from the *An. gambiae* s.s. genome (https://www.vectorbase.org/faqs*)* and their chromosomal locations are shown in Table 2.

**Table 2:** Loci identity showing reference genes, chromosomes of origin, mutated nucleotide and variant codons

SNP ID	Reference gene ID (*An. gambiae*)	Chromosome	SNP type	Reference (*An. gambiae*) codon	Variant codon	Reference amino acid	Variant amino acid	Mutation type
PER-PAS-202	AGAP001856	3R	T/C	GAC	GAU	D	D	S
PER-PAS-082	AGAP001856	3R	C/T	GAU	GAC	D	D	S
PER-PAS-301	AGAP001856	3R	G/A	CGG	CGA	R	R	S
PER-PAS-355	AGAP001856	3R	T/C	GGC	GGU	G	G	S
PER-PAS-370	AGAP001856	3R	G/C	CUC	CUG	L	L	S
CRY1_E04-240	AGAP001958	2R	T/C	CGG	UGG	R	W	NS
CRY1_E04-206	AGAP001958	2R	T/A	UCA	UCU	S	S	S
CRY1_E04-097	AGAP001958	2R	A/G	GAA	AAA	E	K	NS
CRY1_E04-252	AGAP001958	2R	T/C	UUU	UUC	F	F	S
CRY2_E05-351	AGAP004261	2R	C/T	CUU	CUC	L	L	S
CRY2_E05-378	AGAP004261	2R	C/T	CUC	CUU	L	L	S
CRY2_E05-407	AGAP004261	2R	G/C	AGG	AGC	R	S	NS
CRY2_E05-501	AGAP004261	2R	A/G	GCA	ACA	A	T	NS
CRY2_E05-561	AGAP004261	2R	T/C	AAC	AAU	N	N	S
CRY2_E05-045	AGAP004261	2R	T/C	GUU	GUC	V	V	S
CRY2_E05-125	AGAP004261	2R	G/A	GCG	ACG	A	T	NS
CYC_E01-268	AGAP005655	2L	T/C	UGC	UGU	C	C	S
CYC_E01-250	AGAP005655	2L	G/T	UCG	GCG	S	A	NS
CYC_E01-217	AGAP005655	2L	C/A	ACC	CCC	T	P	NS
CYC_E01-093	AGAP005655	2L	G/A	ACG	GCG	T	A	NS
CYC_E01-072	AGAP005655	2L	G/C	CUC	CUG	L	L	S
CYC_E01-021	AGAP005655	2L	T/C	AGU	AGC	S	S	S
CYC_E01-034	AGAP005655	2L	T/C	UUC	UUU	F	F	S
Clk_E01-087	AGAP005711	2L	G/A	GAU	AAU	D	N	NS
Clk_E01-192	AGAP005711	2L	C/T	CUC	UUC	L	F	NS
Clk_E01-240	AGAP005711	2L	G/A	CUG	CUA	L	L	S
Clk_E01-267	AGAP005711	2L	C/T	CAC	UAC	H	Y	NS
PDP1_E02-110	AGAP006376	2L	G/A	GCG	GCA	A	A	S
Vri_E02-355	AGAP007801	3R	C/T	AUC	AUU	I	I	S
Vri_E02-427	AGAP007801	3R	C/T	ACC	ACU	T	T	S
TIM_E05-087	AGAP007801	3R	C/A	CCC	CCA	P	P	S
TIM_E05-189	AGAP007801	3R	A/G	AUG	GUG	M	V	NS
TIM_E05-075	AGAP001856	3R	G/C	GAC	CAC	D	H	NS
TIM_05-195	AGAP007801	3R	C/T	GUC	GUU	V	V	NS

The loci were selected from the 8 clock genes; *PER* period, *CRY1* cryptochrome1, *CRY2* cryptochrome2, *CYC* cycle, *CLK* clock, *Pdp1* par domain protein 1, *VRI* vrille and *TIM* timeless. Mutation type S means synonymous mutation and NS means non-synonymous mutation

All 8 clock genes were PCR amplified in a reaction which included 0.2 X Q solution (QIAgen) 1X buffer (Sigma-Aldrich), 1 mM MgCl, 0.4 mM DNTP, 0.1 mM forward primer, 0.1 mM reverse primer, 1 Unit of HotstarTaq Plus DNA polymerase (QIAgen), ~8 μg/μl DNA and pure water. Successful amplification of PCR products was verified using QIAxcel ScreenGel (QIAgen) software version 1.2. The concentration of DNA was measured by spectrophotometry using NanoDrop 1000 V3.7 (Thermo Fisher Scientific Inc.). Ten microliters of the amplified DNA products were purified using DNASap purification kit and sent for conventional post-PCR Sanger sequencing at a DNA sequencing facility at the University of California Davis. Sanger sequencing was used on a subset of the 64 samples used for the purpose of SNP discovery. Each gene fragment was sequenced in both forward and reverse directions. Sequences were checked for quality control using Geneious software version 6.1 [50], in which manual alignment of forward and reverse strands was conducted. Poor quality sequences were trimmed, and alignment of reads to reference sequences was made.

### SNP genotyping assay and statistical analysis

The Typer Assay Designer software (Agena Biosciences, San Diego, CA) was used to devise a multiplex SNP genotype assay to allow screening of SNP polymorphisms within the samples. These samples were genotyped using the Agena Biosciences MassARRAY iPLEX platform for the full set of 34 loci derived from the 8 clock genes. Negative controls were run for each plate of samples genotyped. A signal to noise ratio of 3 or above the background level was used to call genotypes. The TyperAnalyzer Application (Agena Biosciences, version 4.0.24.71) was used to score genotypes across all 34 loci. The population genetics software DnaSP v.5 [51] was used to identify haplotype sequences from the iPLEX using a phase algorithm to score SNP density, calculate Tajima D statistics, the number of nucleotide substitutions per phenotype based on direct sequencing results, and the number of shared mutations between groups.

The Arlequin software version 3.5 [52] was used to test departure from Hardy-Weinberg equilibrium (HWE) within each locus. The STRUCTURE software [53] was used to conduct clustering analysis to assign populations or individuals into their membership groups based on the feeding behavioural phenotypes (i.e. time and location of feeding). STRUCTURE was run through a burn-in period of 50,000 followed by 50,000 replications. Results from STRUCTURE were uploaded into STRUCTURE Harvester [54] to assess the ∆K statistic according to Evanno [55] in order to select the number of distinct genetic clusters (K) represented within samples. To determine how the *TIM* gene influenced the clustering of samples based on the STRUCTURE, separate STRUCTURE analyses were performed in four groups: the first group included all 8 genes (34 loci), the second group excluded *TIM* (30 loci included), the third group included *TIM* alone (4 loci) and the fourth group involved analysis done on the non-synonymous SNPs alone to sort out the possible noise effect of the synonymous SNPs on the clustering analysis. In all STRUCTURE analyses, eight populations (K = 8) were assumed *a priori*. Finally, IndQsort (http://grassi2.ucdavis.edu/~yoosook/Scripts/indQsort/) was used to reorder individuals according to their membership coefficients. Visualization of population clustering was obtained using the Distruct software [56].

Further confirmation of the effect of *TIM* in the population clustering was assessed by Fisher Exact tests [57] performed by comparing the distribution of the frequencies of each of the 34 loci between the 2 clusters generated by STRUCTURE. To avoid the possibility of obtaining false positive results due to multiple comparisons, a Bonferroni correction [58] was done by dividing the *P*-values by the number of comparisons performed. Additional confirmation of the genetic clustering analysis was done using Principal Component Analysis (PCoA) implemented in GenALEx [59] and available as a plug-in in Excel. PCoA was done for all 34 loci in two steps. Step 1 involved a pull of all 8 phenotypes, while in step 2 each of the 8 phenotypes was analysed separately.

A further study was conducted on published genome sequences of *An. arabiensis* [60] from samples that overlap with our study area (Minepa, Lupiro and Sagamaganga) including an out-group population from Cameroon in West Africa. These genome sequences were mapped to *An. gambiae* mitochondrial sequence ([55]; GenBank ID: NC_002084) using BWA-MEM [61]. Mitochondrial sequences for 24 individual sequences were aligned using Geneious program v.6.1.4. Jukes-Cantor Genetic distance model with bootstrap was used to draw phylogenetic tree.

The 8 SNPs from 8 mitochondrial genes (*ATP6, ATP8, COX1, COX2, COX3, ND3, ND5* and *CYTB*) were selected for SNP genotyping 87 *An. arabiensis* samples from 4 feeding phenotypes (early feeding, late feeding, indoor feeding and outdoor feeding) collected in Sagamaganga and Lupiro. Collection of these samples was done in households using HLC technique. Analysis of these samples followed the same procedures as outlined above. The SNP information including flanking sequences and primer sequences used for iPLEX SNP genotyping are provided in Additional file 1: Table S4. This additional analysis aimed at establishing support for possible population substructure among *An. arabiensis* in the Kilombero valley.

### Ethics statement

The National Institute for Medical Research (NIMR) provided ethical clearance certificate number NIMR/HQ/R.8a/Vol. IX/801 that allowed this research to be conducted. Informed consent forms were prepared by Ifakara Health Institute (IHI) and submitted for approval to the NIMR. Before setting up experiments, household members were informed of the procedures of the experiments and had to read the informed consent forms before participating in the experiments. Willing household owners signed the forms to allow experiments to be done in and around their houses. Volunteers were informed of the procedures to observe during sample collection including risks involved. Participating volunteers were given malaria prophylaxis Malarone (250 mg atovaquone and 100 mg proguanil hydrochloride, GlaxoSmithKline) before and during the experiments to prevent infection.

## Results

All 762 *An. gambiae* s.l. from Lupiro and Sagamaganga villages analysed were *An. arabiensis*. We discovered an average of one SNP in every 46.8 ± 34.5 base-pairs in 8 clock genes, with *CYC* and *VRI* having the highest and the lowest densities of 1 SNP per every 10 and 125 bp respectively. This was comparable to the overall SNP density previously reported for *An. arabiensis* (1 every 47 bp [62]).

### iPLEX SNP genotyping

Twenty-one of the 34 loci genotyped had synonymous mutations, while the remaining 13 loci had non-synonymous mutations (Table 3). SNPs in *TIM* had the highest number of synonymous mutations (ranging from 10 to 15, Table 4) in all the 4 phenotypes. Non-synonymous mutations in *TIM* ranged from 1 to 2, which was notably lower than those reported in the two genes with the highest rates of non-synonymous mutations (e.g. *CRY2* and *CYC* (10–21, Table 4). The remaining genes had low to moderate numbers of synonymous and non-synonymous mutations ranging from 0 to 7 in both cases. No fixed polymorphisms were detected.

**Table 3:** Tajima’s D statistics including synonymous and non-synonymous mutations and nucleotide diversity in 8 clock genes and among early and late feeding phenotypes of *An. arabiensis*

Chr^a^	Gene ID^b^	Gene^c^	Pop^d^	N^e^	n_*s*_^f^	∏(%)^g^	D^h^	μ_*s*_^i^	μ_NS_^j^	μ_NCS_^k^	SNPd^l^	LE:LL^m^	SE:SL^n^	LE:SE^o^	LL:SL^p^	Cod^q^ pos
2L	AGAP005711	Clk E018627	LE	30	12	0.0057	−1.6620	7	4	7	24.31	6	4	7	4	1
			LL	26	7	0.0034	−1.2588	7	0	7						
			SE	30	8	0.0032	−1.5161	8	0	8						
			SL	24	4	0.0032	−0.1632	4	0	4						
2R	AGAP001958	Cry1 E041560	LE	22	8	0.0051	−0.9904	1	7	1	27.36	7	6	7	6	2
			LL	18	10	0.0050	−1.7391	1	9	1						
			SE	28	10	0.0061	−0.9119	1	9	1						
			SL	20	6	0.0054	−0.1223	1	5	1						
2R	AGAP004261	Cry2 E051011	LE	28	16	0.0057	−0.5965	0	16	NA 0	31.31	12	13	15	13	1
			LL	20	15	0.0054	−0.9156	0	15	0						
			SE	36	16	0.0050	−0.7496	0	16	0						
			SL	22	15	0.0054	−0.7795	0	15	0						
2L	AGAP005655	Cyc E018454In5	LE	18	16	0.0138	−0.1541	5	10	5	9.53	13	25	16	23	1
			LL	18	32	0.0301	−0.8217	7	21	7						
			SE	20	26	0.0277	0.5346	6	16	6						
			SL	16	23	0.0198	−0.7512	6	16	6						
2L	AGAP006376	Pdp1 E021381	LE	28	3	0.0021	−1.3214	3	0	3	56.33	0	0	0	0	1
			LL	20	0	NA	NA	NA	NA	NA						
			SE	26	0	NA	NA	NA	NA	NA						
			SL	26	2	0.0009	−1.5131	2	0	2						
3R	AGAP001856	Per PAS	LE	26	5	0.0033	0.0343	0	5	0	68	4	3	4	4	1
			LL	18	5	0.0037	0.1080	0	5	0						
			SE	26	4	0.0036	0.1083	0	4	0						
			SL	20	4	0.0028	0.0781	0	4	0						
3R	AGAP001856	Tim E052569	LE	42	17	0.0073	0.4200	15	2	15	31.82	12	11	11	11	1
			LL	32	12	0.0076	1.1916	10	2	10						
			SE	20	11	0.0057	1.6610	10	1	10						
			SL	22	14	0.0071	0.3339	13	1	13						
3R	AGAP007801	Vri E027461b	LE	28	4	0.0018	−0.4212	0	4	0	125.75	2	3	2	1	3
			LL	18	2	0.0010	0.9062	0	2	0						
			SE	30	3	0.0014	0.2328	0	3	0						
			SL	18	3	0.0014	−0.2589	0	3	0						

^a^Chromosome, ^b^ *An. gambiae* reference gene ID, ^c^Gene, ^e^Number of haplotype sequences, ^f^Number of segregating sites, ^g^Nucleotide diversity, ^h^Tajima’s D, ^i^Number of synonymous mutations, ^j^Number of non-synonymous mutations, ^k^Number of silent mutations, ^l^SNP density i.e. number of SNPs found in a given number of nucleotide base-pairs, ^m^shared polymorphisms between early and late in Sagamaganga, ^n^shared polymorphisms between early and late in Sagamaganga, ^o^shared polymorphisms in early biting between Lupiro and Sagamaganga, ^p^shared polymorphisms in late biting between Lupiro and Sagamaganga, and ^q^Codon at which the SNP occurs

**Table 4:** STRUCTURE assignment of allele frequencies of clock gene SNPs

Locus	SNP	Cluster 1	Cluster 2	Locus	SNP	Cluster 1	Cluster 2	Locus	SNP	Cluster 1	Cluster 2
clk-e01-087	G	0.957	0.952	cyc-e01-072	C	0.099	0.094	cry2-e05-378	C	0.883	0.889
	A	0.043	0.048		G	0.901	0.906		T	0.117	0.111
clk-e01-192	C	0.865	0.890	cyc-e01-093	G	0.461	0.503	cry2-e05-407	T	0.106	0.082
	T	0.135	0.110		A	0.539	0.497		C	0.894	0.918
clk-e01-267	T	0.847	0.900	cyc-e01-034	T	0.239	0.183	cry2-e05-501	G	0.088	0.088
	C	0.153	0.100		C	0.761	0.817		A	0.912	0.912
clk-e01-240	G	0.933	0.933	cyc-e01-217	C	0.931	0.893	cry2-e05-561	C	0.975	0.971
	A	0.067	0.067		A	0.069	0.107		T	0.025	0.029
cry1-e04-097	C	0.938	0.946	cyc-e01-021	T	0.567	0.630	cry2-e05-051	T	0.913	0.911
	T	0.062	0.054		C	0.433	0.370		C	0.087	0.089
cry1-e04-206	G	0.912	0.917	cyc-e021-250	G	0.119	0.096	cry2-e05-045	C	0.421	0.407
	A	0.088	0.083		T	0.881	0.904		T	0.579	0.593
cry1-e04-240	G	0.969	0.968	cyc-e01-268	T	0.481	0.471	cry2-e05-125	G	0.859	0.888
	A	0.031	0.032	-	C	0.519	0.529		A	0.141	0.112
cry1-e04-252	G	0.771	0.764	-	-	-	-	cry2-e05-351	T	0.533	0.528
	A	0.229	0.236	-	-	-	-		C	0.467	0.472
per-pas-082	C	0.929	0.931	tim-e05-189	G	0.513	0.022	pdp1-e02-110	G	0.967	0.977
	T	0.071	0.069		A	0.487	0.978	-	A	0.033	0.023
per-pas-202	C	0.643	0.624	tim-e05-495	G	0.764	0.991	-	-	-	-
	T	0.357	0.376		A	0.236	0.009	-	-	-	-
per-pas-355	C	0.799	0.829	tim-e05-075	G	0.570	0.994	vri-e02-355	C	0.978	0.987
	T	0.201	0.171		C	0.430	0.006		T	0.022	0.013
per-pas-370	C	0.646	0.569	tim-e05-087	C	0.187	0.489	vri-e02-427	C	0.974	0.963
	G	0.354	0.431		A	0.813	0.511		T	0.026	0.037

The *Timeless* gene was associated with the binary clustering into clusters 1 and 2

**Fig. 2:**
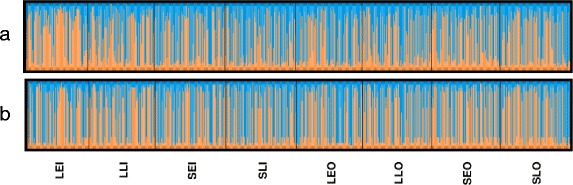
STRUCTURE clustering results with parameter K = 2. All 34 loci icluded in (**a**), and in (**b**) only the 4 *TIM* loci included. LEI=Lupiro early indoors, LLI=Lupiro late indoors, SEI=Sagamaganga early indoors, SLI=Sagamaganga late indoors, LEO=Lupiro early outdoors, LLO=Lupiro late outdoors, SEO=Sagamaganga early outdoors, SLO=Sagamaganga late outdoors. There appears to be two population sub-divisions (*blue and yellow*) across samples with different feeding behaviours

There was no evidence of genetic distance between *An. arabiensis* samples from different feeding locations (indoor vs outdoor, F_ST_ <0.001), feeding times (early vs late, F_ST_ <0.001), or geographical locations (Sagamaganga vs Lupiro, F_ST_ <0.001). None of the Tajima’s D values were significant (Table 3), indicating the SNPs are evolving neutrally with no evidence of selection, demographic expansion or contraction. All SNPs were in Hardy-Weinberg equilibrium, suggesting an absence of selection on these candidate genes within the populations. STRUCTURE assigned individuals into two distinct groups based on observed patterns of genetic variation (Fig. 2). However, this substructure was not associated with feeding behaviour or site of collection. Analysis of the membership coefficients of cluster 1 and 2 revealed that the frequencies of SNPs in *TIM* were most divergent between two clusters (Table 4). Fisher Exact tests on the frequencies of all the 34 loci between cluster 1 and 2 showed that there was no difference in the frequency of 30 loci distributed between cluster 1 and 2, while the 4 loci belonging to *TIM* showed strong significant difference (*P* < 0.0001) in their distribution among cluster 1 and 2 following Bonferroni correction (Table 5) implying that *TIM* strongly drives the binary clustering observed in the samples.

**Table 5:** Significance test for Fisher Exact test between cluster 1 and 2 as assigned by STRUCTURE

Gene	Number of loci	Number of fisher exact comparisons performed between cluster 1 and 2	*P*-value (after Bonferroni correction)
*Clk*	4	8	0.123
*Cry1*	4	8	0.125
*Per*	4	8	0.122
*Cyc*	7	14	0.071
*Tim*	4	8	<0.0001*
*Cry2*	8	16	0.063
*Pdp1*	1	2	0.500
*Vri*	2	4	0.333

*P*-values are for the significance test for the association of the frequency of SNPs between cluster 1 and 2 following Bonferroni correction

Further analysis of the clustering results from STRUCTURE was carried with the aim of determining the effect of *TIM* on the population structure of *An. arabiensis* in the study site. Analysis of clustering results from STRUCTURE performed on all 34 loci including the 4 loci from *TIM* gave a moderate support for 2 clusters irrespective of the feeding phenotypes and origin of the sample (Additional file 2: Figure S1A). Support for K = 2 was stronger when loci from *TIM* alone were analysed separately (Additional file 2: Figure S1B) suggesting that *TIM* played a key role in the binary structuring of *An. arabiensis*.

**Fig. 3:**
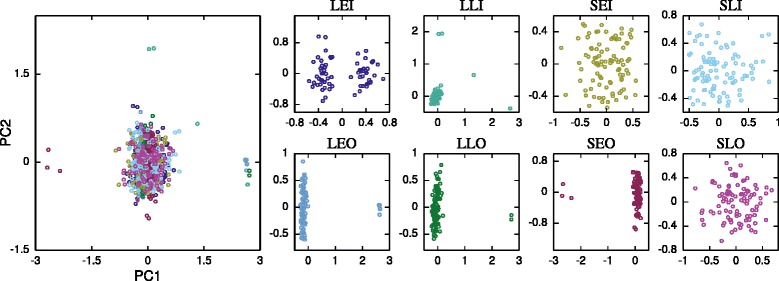
Principal Component Analysis based on the genetic distances generated by STRUCTURE at K = 2*.* All 8 phenotypes were combined in one analysis and separate analysis for each of the 8 feeding phenotypes. Coordinate 1 and 2 represent the first and second principal components, respectively. PCoA analysis included a total of 730 samples from 8 feeding phenotypes from Lupiro and Sagamaganga: LEI (Lupiro early indoors), LEO (Lupiro early outdoors), LLI (Lupiro late indoors), LLO (Lupiro late outdoors), SEI (Sagamaganga early indoors), SEO (Sagamaganga early outdoors), SLI (Sagamaganga late indoors), SLO (Sagamaganga late outdoors). All 34 loci were included in the PCoA analysis

Principal Component Analysis (PCoA) results of all 8 phenotypes combined showed that there was no population sub-division based on the feeding phenotypes (Fig. 3), confirming the same results obtained from the STRUCTURE analysis. PCoA revealed two sub-populations more pronounced in samples from Lupiro compared to those from Sagamaganga (Fig. 3). This suggests presence of two sub-populations irrespective of the feeding behaviour of mosquitoes in Lupiro and Sagamanga villages. A geographical association in the sub-structuring of *An. arabiensis* is yet to be established.

### Further support for population sub-structuring in the Kilombero valley

**Fig. 4:**
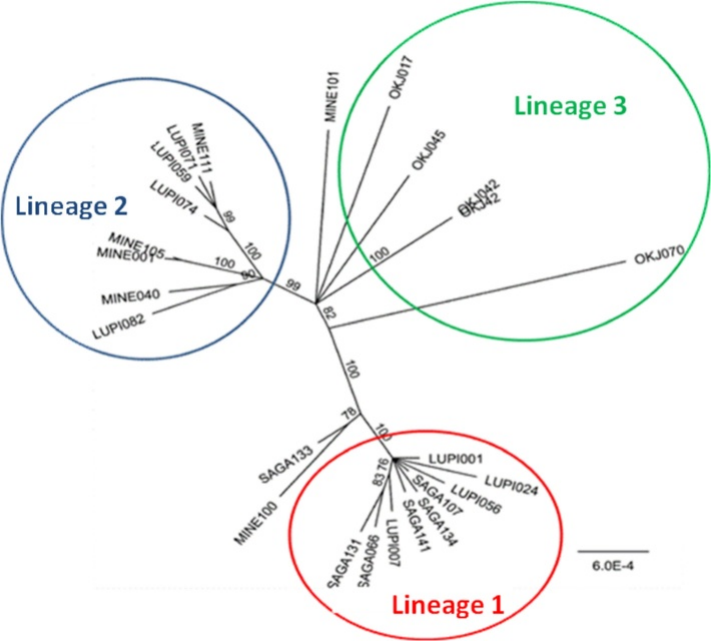
Unrooted tree based on Jukes-Cantor Genetic distance model with bootstrap using complete mitochondrial sequences extracted from [60]. Three distinct groups were identified: Sagamaganga group which included samples from Sagamaganga and Lupiro, Minepa group with samples from Minepa and Lupiro and the Cameroon group

We further investigated the population structure within *An. arabiensis* in our study site using the published genome sequence data of *An. arabiensis,* which overlap with our study area of Lupiro and Sagamaganga [60]. We identified fixed nucleotide differences between Minepa and Sagamaganga lineages in 7 out of 8 SNPs from 8 mitochondrial genes (Additional file 3: Table S2). We also found two major genetic clusters in samples from the Kilombero valley and those from Cameroon based on the mitochondrial sequences (Fig. 4). Analysis revealed samples from the Kilombero valley to be subdivided into two major lineages. The two major lineages are Lineage 1 that includes samples from Sagamaganga, Minepa and Lupiro, and Lineage 2 that includes samples from Minepa and Lupiro (Fig. 4). Samples from Cameroon and one from Minepa clustered out in a separate group (Lineage 3, Fig. 4). These results indicate that Tanzanian population of *An. arabiensis* is subdivided, as was suggested in [63]. Subdivision within Tanzania appears to be somewhat geographically related. However, from the 87 samples from four feeding phenotypes from Lupiro and Sagamaganga that were genotyped, two lineages were found in both sites regardless of the phenotype group (Additional file 4: Table S3). The reproductive isolation between two lineages has yet to be determined. Overall the genetic clusters based on mitochondrial sequence supports that there are at least two populations of *An. arabiensis* in our study site that are not related geographically nor according to biting time differences.

## Discussion

This study investigated genetic diversity within *An. arabiensis* with the aim of assessing whether variation in a set of eight clock genes, which critical components of the molecular oscillator controlling rhythmic behaviour, could explain differences in the time and location (indoors *versus* outdoor) of their host-seeking behaviour. In total, 34 loci incorporating 313 polymorphic sites were assayed. Based on this subset of genes, there was no evidence that the feeding behavioural phenotypes (early *vs* late and indoor *vs* outdoor feeding) of *An. arabiensis* clustered as distinct genetic subpopulations.

There may be several reasons for this lack of association. First, it should be recognised that the molecular oscillator controlled by clock genes is still poorly understood in haematophagous insects, in contrast to *Drosophila* species [47]. Whilst the candidate clock genes used here were drawn from studies in *Drosophila* and have been shown to have time-dependent expression in *An. gambiae* (*s.s*.) [41], their mode of action and daily expression patterns in relation to rhythmic activities in *An. arabiensis* have not yet been confirmed. Additionally, lack of correlation of SNPs in core clock genes and timing of biting behaviour highlights the possibility that alterations in feeding rhythms could be due to changes in the neuronal network properties and/or activity that connects the molecular oscillator to output mechanisms that control feeding. Finally, failure to link genetic mutations to feeding behaviour phenotypes in *An. arabiensis* could be due to methodological issues, including the usage of too few markers, as linkage disequilibrium in *An. arabiensis* has been shown to breakdown within 200 bp [60]. Other reasons could be, use of inappropriate markers, imprecise classification of phenotypes and/or the fact that extensive phenotypic plasticity in feeding behaviour is possible within one genotype [64]. Use of individuals from the extreme of the time continuum, e.g. those that bite during the day and those that bite late at night, could increase chances of finding association between feeding and genetic structure. However, this is practically impossible as *An. arabiensis* are not known to feed during the day, making it possible only to sample mosquitoes which feed during the night.

A potential limitation of this study, which may have reduced the ability to identify clear phenotype-genotype associations, was that the biting time phenotypes were quite coarsely and perhaps imprecisely defined. Selection of phenotypes in this study was based on broad categorisation into “early” *vs* “late” feeding groups, with each period spanning 3 h of collection. Though such categorisation was based on evidence from previous studies which showed that some mosquitoes prefer to feed early at dawn while some feed late at night [15, 65–67], it is not evident that the two phenotypes represent two genetically different groups or just the same group feeding at two different periods of the night. Further studies involving finer scale timing of the feeding behaviour, for example within an hourly interval could be tested in the future. Another potential imprecision is that the time at which mosquitoes were collected may not necessarily have reflected the time at which they initiated their host-seeking. For example, those caught during the late period may actually have begun feeding during the early period of the night, but been unsuccessful in locating a host. Other factors such as distance from host, wind direction, wind speed, and availability of other hosts were not discussed, because all the samples used in this analysis were collected within two days period in each location. The availability of other hosts was different between two locations, Lupiro and Sagamaganga, but we did not detect any difference in feeding time profile between two locations. It would be difficult to assess this under natural conditions, but more detailed investigation of a small number of mosquitoes under lab or semi-field settings may be viable.

Finally, although the clock genes investigated here were not linked with feeding behaviours, variation noted here may be associated with other behaviours which influence gene flow. Specifically the clustering of mosquito population into two groups by the *TIM* in this study may indicate presence of two coexisting populations that have mating incompatibility possibly through temporal and cytological incompatibility.

Whilst behavioural phenotypes showed no genetic basis here, there was evidence of strong genetic clustering within *An. arabiensis* samples in association with *TIM*. The association with *TIM* was so strong that the same pattern of genetic structure was predicted from this gene alone as with all eight clock genes combined (Additional file 2: Figure S1). Studies have shown that *TIM* is involved in regulating circadian rhythms, which may influence the time of feeding [39, 68]. *TIM* has also been found to regulate autophagy and diapause [68]. Furthermore, markers based on *TIM* alone have been used to identify population structure in *Anopheles cruzii* Dyar & Knab [69], and in *Anopheles triannulatus* s.l. (Neiva & Pinto) [70]. Additionally, *TIM* has also shown variation in expression levels across different times of day in the pitcher-plant mosquito *Wyeomyia smithii* (Coquillett) [71]. The link between *TIM* and population structure in these studies suggests that this gene may also be playing a role in population structure of *An. arabiensis.*

While it may not be surprising to find samples from different feeding phenotypes and from the 40 km apart villages of Lupiro and Sagamaganga showing no genetic clustering, it is however interesting for samples within the villages to cluster based on *TIM*. Studies involving other insects have shown population clustering based on *TIM* across geographical locations. For example, in a study of frequencies of allele (*ls-tim*), which is one of two alleles of *TIM* in *Drosophila* species, variation of frequencies of this allele was shown across geographical location between Italy, Israel and Zimbabwe [68]. In our study, it remains puzzling that *TIM* showed population clustering within a village but not across villages. This may suggest genetic linkage between *TIM* and genes controlling mating incompatibility through cytological and temporal incompatibility of *An. arabiensis* population within a locality. It may be interesting to investigate how different alleles of *TIM* cluster across different geographical locations in the Kilombero valley to have a broader insight of the population structure of *An. arabiensis* within and between geographical localities. Further analysis based on larger set of markers or whole genome is required to unambiguously confirm of the effect of *TIM* on the observed clustering. However the existence of such fine-scale genetic structure as revealed by even the limited number of markers used here indicates there may be natural barriers to geneflow within *An. arabiensis* occurring over very small distances in the Kilombero Valley.

A few previous studies have investigated the population genetics of *An. gambiae (s.l.)* within the Kilombero Valley. One study similarly revealed the presence of strong structuring within *An. arabiensis* at the village-level in the Kilombero Valley, indicating this species exists in genetically distinct populations between villages situated only 40 km apart [63]. However in a recent study [72], *An. arabiensis* from the Kilombero Valley were predicted to exist within a single population including others from coastal Tanzania and the islands of Zanzibar. Further, a previous continental analysis of *An. arabiensis* population structure predicted relatively high levels of gene flow even between populations situated more than a 1000 km apart [73]. These contrasting findings may be partially due to limitations and discrepancies due to variation in the methods of analysis and selection of markers used.

Additional analyses of *An. arabiensis* population structure in our study using SNPs from mitochondrial genes has strengthened our arguments and those from previous work [63] indicate that *An. arabiensis* in the Kilombero valley may be divided into multiple subpopulations. While SNPs analysis from the clock genes showed possibility for two sub-populations of *An. arabiensis* that are not geographically related, analysis of mitochondrial SNPs has shown distinctive population structure within villages that are just 25 km apart (Fig. 4). These results in common strengthen the argument for sub-structure in *An. arabiensis,* which could be within or between localities in the Kilombero valley. This is an interesting phenomenon that requires further investigation using a broad range of markers to better understand the evolutionary processes in these vectors.

Future studies on the genetics of *An. arabiensis* behaviour should ideally involve a broader sampling strategy both with respect to the range and resolution of phenotypes selected, and the number of SNP markers used to increase the possibility of detecting genetic influences on their time and location of blood feeding. Further, it is possible that candidate clock genes may influence *An. arabiensis* feeding behaviour indirectly * via* epistatic interactions with other genes not considered here. To increase the power to detect both direct and epistatic genetic impacts on *An. arabiensis,* future studies using whole genome sequencing approaches are recommended. Additionally, transcriptomic approaches may also be useful given that they have been used successfully to detect associations between host choice behaviours and gene regulation in other insect vectors [74].

## Conclusions

This study did not find any association between feeding behavioural phenotypes (early *vs* late and indoor *vs* outdoor feeding) in *An. arabiensis* and single nucleotide polymorphisms identified from eight candidate clock genes. However, there was evidence that the population contained two distinct genetic clusters that were associated with the *Timeless* gene, independently of feeding phenotype or geographical location. This finding was supported by analysis of eight mitochondrial genes, which showed that two lineages exist between two *An. arabiensis* populations just 25 km apart. It is highlighted that investigations of the genetic basis of the feeding behaviour in malaria vectors are still in their infancy, and will likely require much further development through use of high-resolution markers distributed across the entire genome, and/or the application of other methods including transcriptomic approaches to provide a strong test of genotype-phenotype associations. In studies where markers covering the whole genome have been applied, the high density and short LD in *An. arabiensis* observed implies that huge sample sizes could be needed to robustly test for such associations, which at present are not viable due to the high cost and time requirements. These limitations make a candidate gene approach more attractive in the short-term, however in future we recommend this could be improved through use of a larger set of genes, selected from across the entire genome of malaria vectors.

## Additional files

Additional file 1: Table S4. The SNP information including flanking sequences and primer sequences used for iPLEX SNP genotyping. (XLSX 13 kb) Additional file 2: Figure S1. Bayesian clustering analysis*.* The magnitude of ΔK as a function of K showing K = 2 as the most probable number of clusters when all 34 loci were included (A) and when only 4 *Timeless* loci were included in analysis (B). In (C) analysis was done on the remaining 30 with the exclusion of the *Timeless* loci. In D only the non-synomymous SNPs were analys ed. Assumption of 8 populations was made *apriori.* The 8 populations were assumed according the 8 feeding phenotypes, which included 4 phenotypes from Lupiro and 4 from Sagamaganga (PDF 1039 kb) Additional file 3: Table S2. Nucleotide sequences on the 8 genes from mitochondria that distinguish two lineages (MINEPA and SAGAMAGANGA) in Tanzania. (DOCX 11 kb) Additional file 4: Table S3. Sample size and Chi-square test results between the feeding phenotypes and the sampling sites of Lupiro and Sagamaganga. (DOCX 11 kb)
