# Supplementary figures and images for: Investigating associations between biting time in the malaria vector Anopheles arabiensis Patton and single nucleotide polymorphisms in circadian clock genes: support for sub-structure among An. arabiensis in the Kilombero valley of Tanzania
Source: Parasit Vectors. 2016 Feb 27;9:109. doi: 10.1186/s13071-016-1394-8 (PMC4769569; doi:10.1186/s13071-016-1394-8)

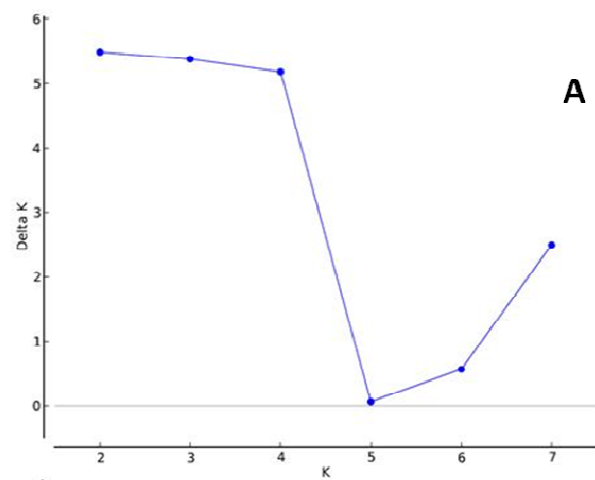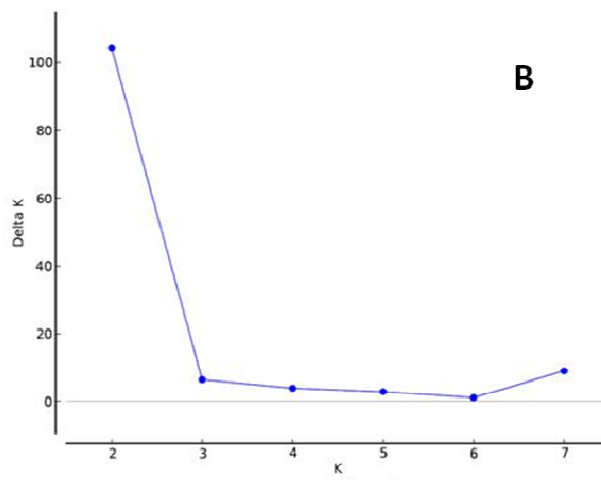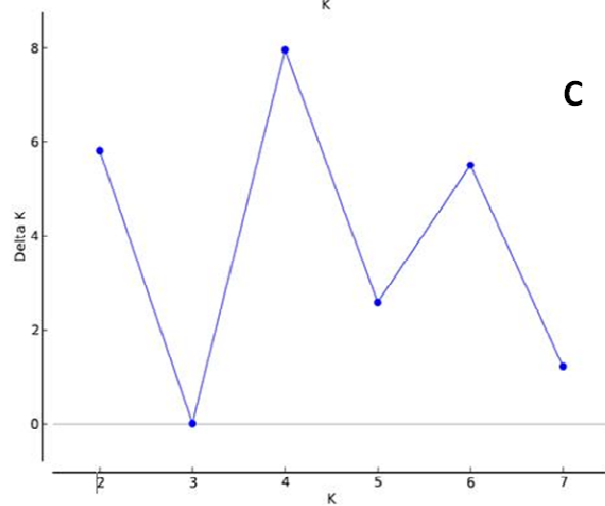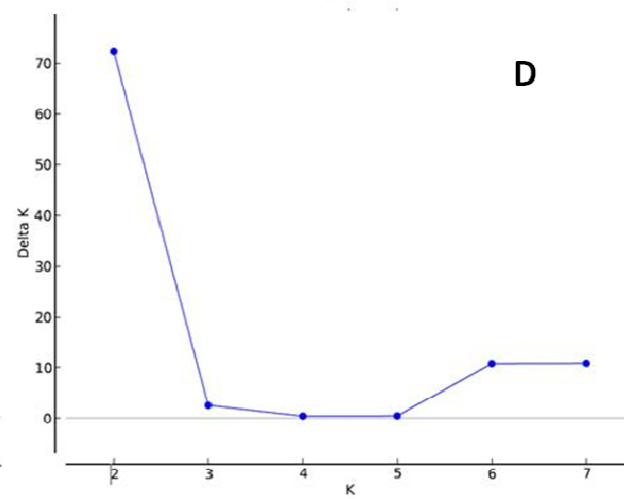

Supplement: Additional file 2: Figure S1. — Bayesian clustering analysis. The magnitude of ΔK as a function of K showing K = 2 as the most probable number of clusters when all 34 loci were included (A) and when only 4 Timeless loci were included in analysis (B). In (C) analysis was done on the remaining 30 with the exclusion of the Timeless loci. In D only the non-synomymous SNPs were analys ed. Assumption of 8 populations was made apriori. The 8 populations were assumed according the 8 feeding phenotypes, which included 4 phenotypes from Lupiro and 4 from Sagamaganga (PDF 1039 kb) [file 13071_2016_1394_MOESM2_ESM.pdf]
