# Supplementary material for: Investigating associations between biting time in the malaria vector Anopheles arabiensis Patton and single nucleotide polymorphisms in circadian clock genes: support for sub-structure among An. arabiensis in the Kilombero valley of Tanzania
Source: Parasit Vectors. 2016 Feb 27;9:109. doi: 10.1186/s13071-016-1394-8 (PMC4769569; doi:10.1186/s13071-016-1394-8)
Supplement: Additional file 3: Table S2. — Nucleotide sequences on the 8 genes from mitochondria that distinguish two lineages (MINEPA and SAGAMAGANGA) in Tanzania. (DOCX 11 kb) [file 13071_2016_1394_MOESM3_ESM.docx]

|  | Gene | COX1 | COX2 | ATP8 | ATP6 | COX3 | ND3 | ND5 | CYTB |
| --- | --- | --- | --- | --- | --- | --- | --- | --- | --- |
| Sample | Group | COX  COX-2199 | COX-3447 | ATP8-3860 | ATP6-4105 | COX3-4959 | ND3-5861 | ND5-7618 | CYTB-11442 |
| 2013SLO193 | MINEPA | A | A | T | G | C | T | G | T |
| 2013LEI032 | SAGAMAGANGA | G | G | C | A | A | C | A | C |
