# Supplementary material for: Investigating associations between biting time in the malaria vector Anopheles arabiensis Patton and single nucleotide polymorphisms in circadian clock genes: support for sub-structure among An. arabiensis in the Kilombero valley of Tanzania
Source: Parasit Vectors. 2016 Feb 27;9:109. doi: 10.1186/s13071-016-1394-8 (PMC4769569; doi:10.1186/s13071-016-1394-8)
Supplement: Additional file 4: Table S3. — Sample size and Chi-square test results between the feeding phenotypes and the sampling sites of Lupiro and Sagamaganga. (DOCX 11 kb) [file 13071_2016_1394_MOESM4_ESM.docx]

|  | LUPIRO | SAGA |  | EARLY | LATE |  | INDOOR | OUTDOOR |
| --- | --- | --- | --- | --- | --- | --- | --- | --- |
| MIN | 18 | 26 |  | 17 | 27 |  | 21 | 23 |
| SAG | 13 | 17 |  | 15 | 15 |  | 15 | 15 |
| Undetermined | 9 | 4 |  | 9 | 4 |  | 7 | 6 |
| Chi-square test group | Lupiro vs Sagamaganga | |  | Early vs late biting | |  | Indoor vs outdoor biting | |
| P value | 0.1855 | |  | 0.1408 | |  | 0.9248 | |
